# A Coronary Conundrum: Papillary Muscle Rupture and Ischemic Mitral Regurgitation Secondary to Coronary Thromboembolism in Antiphospholipid Syndrome

**DOI:** 10.1177/2324709619842247

**Published:** 2019-04-22

**Authors:** Amar Shere, Pradyumna Agasthi, Farouk Mookadam, Sudheer Konduru, Reza Arsanjani

**Affiliations:** 1 Mayo Clinic Arizona, Phoenix, AZ, USA

**Keywords:** antiphospholipid syndrome, papillary muscle rupture, ischemic mitral regurgitation, coronary thromboembolism

## Abstract

Antiphospholipid syndrome (APS) is an autoimmune disorder that has a strong propensity for a hypercoagulable state and is known to be associated with venous and arterial thromboembolism. We describe an uncommon case of APS in the setting of non-Hodgkin’s lymphoma, with thromboembolism, and a rare complication after an uncommon etiology of myocardial infarction. This case highlights the importance of early and appropriate type of anticoagulation to reduce the morbidity and mortality in patients with APS.

## Introduction

Antiphospholipid syndrome (APS) is an autoimmune disorder that manifests as a hypercoagulable state typically classified as either a primary disease or secondary to an underlying autoimmune disease, hematologic disorder, or solid tumor.^1^ High levels of antiphospholipid antibodies (aPL) in APS are associated with cardiac valvular lesions and thromboembolic events.^2^ In this article, we observed a patient with APS and non-Hodgkin’s lymphoma develop non-ST elevation myocardial infarction (NSTEMI) with eventual papillary muscle rupture and severe mitral regurgitation (MR) in the setting of a coronary thromboembolism from a nonbacterial thrombotic endocarditis.

## Case Presentation

**Figure 1.**
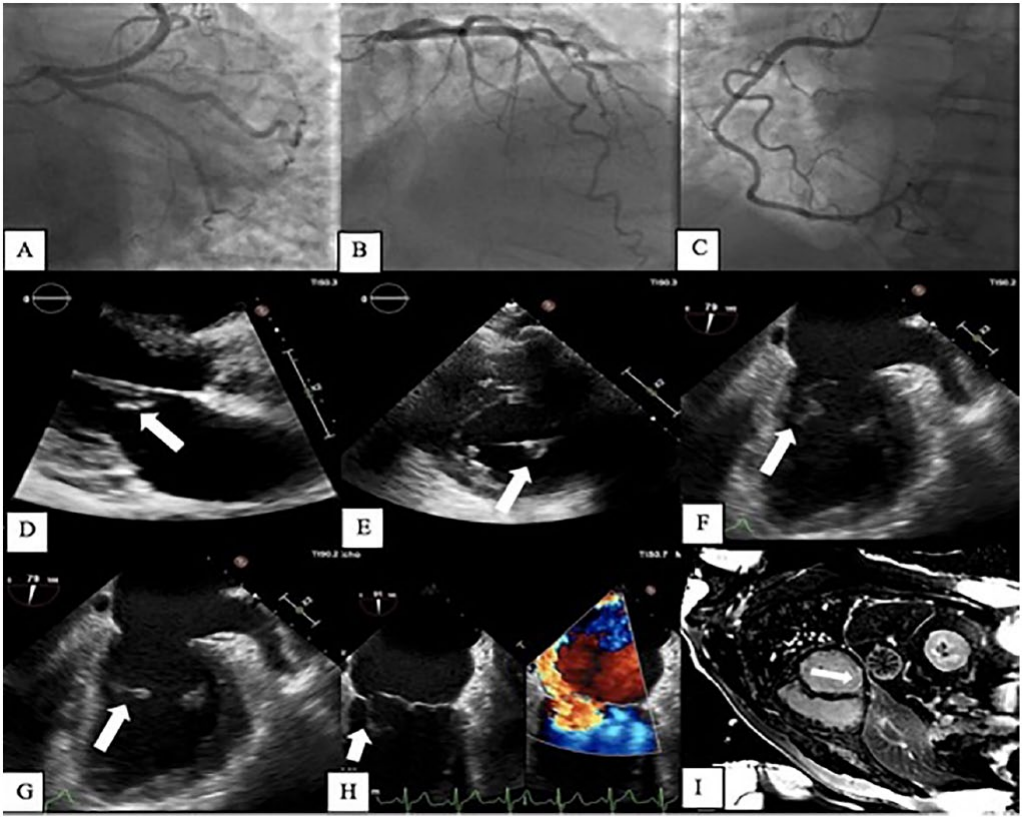
(A-C) Cardiac catheterization displaying nonobstructive coronary artery disease. (D) TTE parasternal long axis (PLAX) view of mitral value displaying flail A2/A3 segment (arrow). (E) Parasternal short axis (PSAX) view displaying ruptured posteromedial papillary muscles (PMPM; arrow). (F and G) TEE displaying ruptured PMPM (arrow). (H) TEE displaying severe mitral regurgitation with a posterior medially directed jet due to ruptured PMPM (arrow). (I) CMR short axis view demonstrating subendocardial late gadolinium enhancement involving basal to mid-inferolateral wall (arrow).

A 52-year-old gentleman with a history of deep venous thrombosis, APS, and diffuse B-cell type non-Hodgkin’s lymphoma presented to the hospital for evaluation of skin necrosis. Three months prior, he had an episode of NSTEMI. Coronary angiogram showed no significant atherosclerotic disease (Figure 1A-C). Direct oral anticoagulation was discontinued, which had been initiated for treatment of a deep venous thrombosis, prior to his NSTEMI. He was symptomatic with dyspnea prior to presentation and was noted to have progressively worsening dyspnea on exertion and rest during the hospital course.

On admission, the patient was afebrile, heart rate 91 beats/min, blood pressure 122/77 mm Hg, respiratory rate 18 breaths/min, and an oxygen saturation of 95% on 1.5 liters by nasal cannula. Physical examination revealed a significantly elevated jugular venous distention measuring approximately 15 cm, with a positive hepatojugular reflux. Auscultation revealed a grade 4/6 holosystolic murmur, heard best at the left ventricular apex, with an S3 gallop. There was trace pitting edema bilaterally, along with extensive necrotic skin lesions across the anterior chest and abdominal wall. Electrocardiogram showed normal sinus rhythm at 95 beats per minute with left anterior fascicular block.

A transthoracic echocardiogram (TTE) showed severe MR with a flail A2/A3 mitral valve leaflet and an eccentric and posteriorly directed MR jet, and mitral regurgitant volume was 73 mL/beat. The left ventricular ejection fraction of 42% with a significantly elevated right ventricular systolic pressure of 85 mm Hg (Figure 1D and E). A transesophageal echocardiogram (TEE) demonstrated a flail A2/A3 segment of mitral valve and a ruptured posteromedial papillary muscle (Figure 1F and G). Severe MR was present, which was posteromedially directed along with pulmonary venous systolic flow reversal consistent with severe MR (Figure 1H). There was no evidence of vegetation or intracardiac thrombus.

Cardiac magnetic resonance imaging (CMR) demonstrated subendocardial late gadolinium enhancement involving basal to mid-inferolateral wall of the left ventricle (Figure 1I). The study also confirmed severe primary MR secondary to ruptured posterior papillary muscle and flail mitral valve leaflets. The findings were consistent with possible prior ischemic event due to a cardioembolic phenomenon resulting in papillary muscle rupture and subsequent severe valve regurgitation.

Laboratory markers were positive for aPL and β-2 glycoprotein I antibody (β-2GPI) consistent with antiphospholipid syndrome. Antiphospholipid antibody immunoglobulin (Ig) M and β-2GPI IgM were both more elevated at a value of 28 and 22.2 MPL (IgM Phospholipid Units) than IgG levels of 9.8 and 9.4 GPL (IgG Phospholipid Units), respectively. Guideline-directed medical therapy was initiated, and the patient subsequently received a 31/33 On-X mechanical mitral valve (CryoLife Inc, Kennesaw, GA) replacement. He was discharged on lifelong warfarin therapy along with aspirin. One year postoperatively, he is currently New York Heart Association (NYHA) functional class I.

## Discussion

Antiphospholipid syndrome is an autoimmune disorder in which patients are at high risk of developing a hypercoagulable state leading to thrombosis.^2^ The presence of aPL in APS has a greater risk of developing thromboembolism and cardiac valvular disease.^3^ Although there have been reports of antibody immune complex deposition causing valvular damage and insufficiency in most APS patients, MR after a non-atherosclerotic myocardial infarction is rare. Patients with APS experience a higher incidence of arterial compared with thromboembolic events.^4^ Thrombotic events are typically seen in solid tumor malignancies rather than lymphoid-derived neoplasm.^5^

Cardiac manifestations of APS have been widely recognized including valvular abnormalities, occlusive arterial diseases, intracardiac emboli, and coronary thromboembolism.^6^ Miranda and colleagues reported a case of thrombosis of the anterior descending artery as a clinical complication of APS in a patient presenting with acute myocardial infarction.^7^ Another case by Park and colleagues demonstrated a patient with systemic lupus erythematosus and APS who developed an inferior myocardial infarction that was complicated by severe MR caused by posterior papillary muscle rupture.^8^ The provoking factors in many of these cases involve presence of aPL, which are directly involved in the pathogenesis of thrombosis. The aPL have been reported to be positive in 11% of cases with myocardial infarction.^9^ In our patient, the cardiac MR results were consistent with subendocardial infarction in the basal to mid-inferior/inferolateral wall of the left ventricle most likely from an embolus from the nonbacterial thrombotic endocarditis, which in turn was the result of APS.

Furthermore, our patient was not anticoagulated at the time of these events, increasing the risk of developing thrombosis. The risk of thromboembolic events significantly increases in patients positive for APS antibodies like β-2GPI; therefore, prolonged anticoagulation is recommended. The first-line guideline therapy by the 14th International Congress on Antiphospholipid Antibodies Task Force Report on APS treatment trends for patients with thrombotic APS is with vitamin K antagonists (VKAs) like warfarin.^10^ Management with VKAs in patients with APS can be challenging because of their multiple food and drugs interactions, which necessitates frequent monitoring of adequate anticoagulation. The variable response of thromboplastin reagents to aPL may potentially influence the validity of prothrombin time/international normalized ratio, thus complicating monitoring.^11^

The rivaroxaban in antiphospholipid syndrome randomized controlled trial suggests that rivaroxaban has the potential to be a convenient and effective alternative to warfarin in thrombotic APS patients requiring standard anticoagulation therapy.^12^ However, its use in high-risk patients with antiphospholipid syndrome has been shown to be associated with increased rates of adverse effects with no benefit.^13^ A clinical trial by Pengo and colleagues demonstrated a 12% rate of thromboembolic events in patients randomized to rivaroxaban compared with 0% in the warfarin group.^13^ The role of direct oral factor Xa antagonists in APS and other inherited thrombophilias remains unclear, and further studies on large cohorts are needed to establish the safety and efficacy of its use over traditional VKAs.

In our patient, coronary thromboembolism most likely contributed to the valvular lesions of non-thrombotic bacterial endocarditis, which is seen in patients with acquired APS. Subsequent embolism down the right coronary artery resulting in posterior circulation myocardial infraction, with papillary muscle rupture, and ensuing severe MR was the etiology for the patient’s symptoms. Multimodality imaging with TTE, TEE, and CMR confirmed these findings. Optimal long-term anticoagulation based on guidelines is with VKAs. Early and appropriate anticoagulation must be made soon after diagnosis to reduce morbidity and mortality in patients with APS.
